# Supplementary figures and images for: Impact of Genomic Mutation on Melanoma Immune Microenvironment and IFN-1 Pathway-Driven Therapeutic Responses
Source: Cancers (Basel). 2024 Jul 17;16(14):2568. doi: 10.3390/cancers16142568 (PMC11274745; doi:10.3390/cancers16142568)

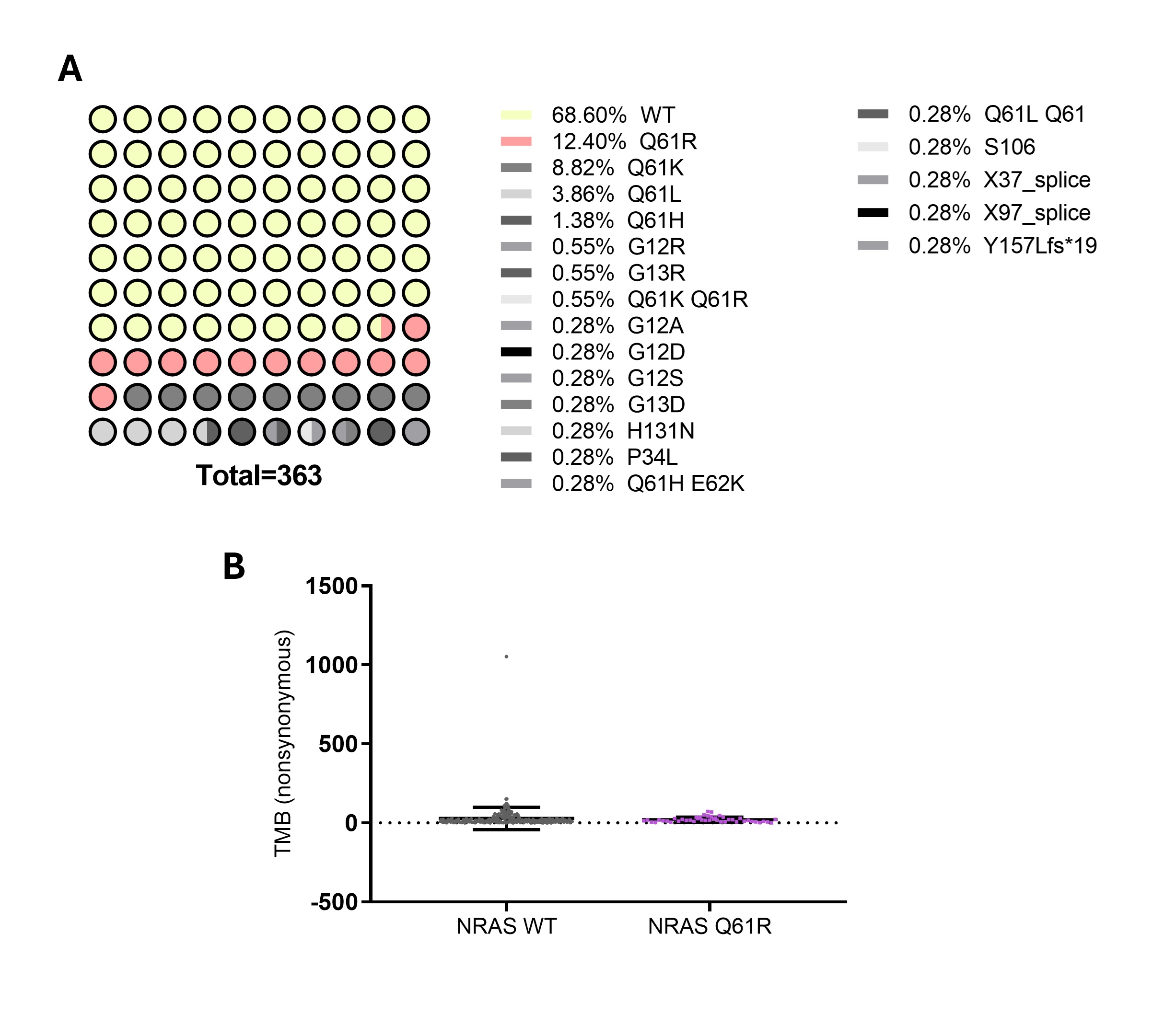

Supplement: Supplementary file 1 [file cancers-16-02568-s001.zip › Figure 1 Supp.jpg]

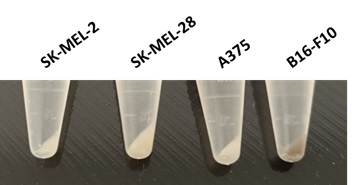

Supplement: Supplementary file 1 [file cancers-16-02568-s001.zip › Figure 2 Supp.jpg]

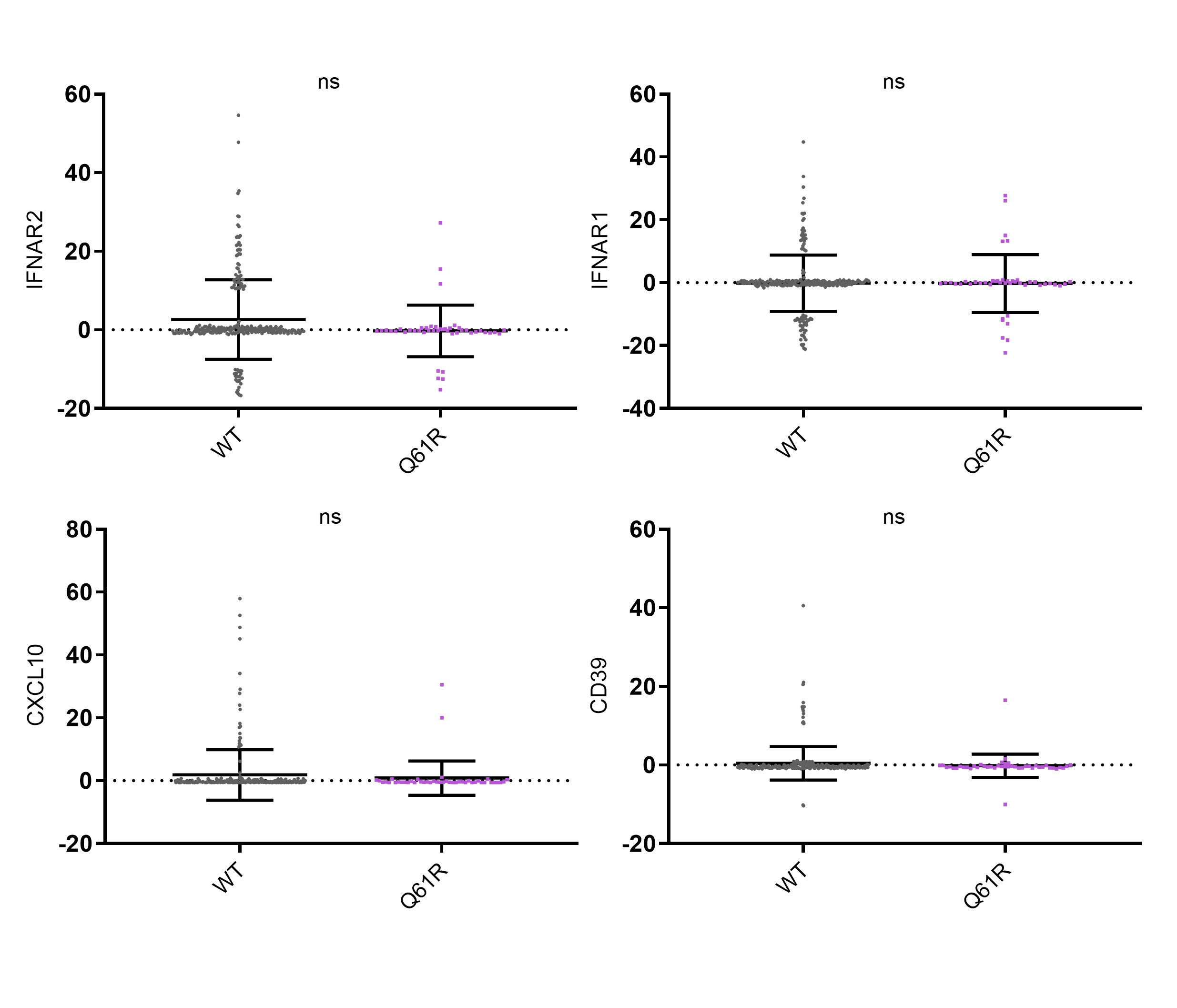

Supplement: Supplementary file 1 [file cancers-16-02568-s001.zip › Figure 3 Supp.jpg]

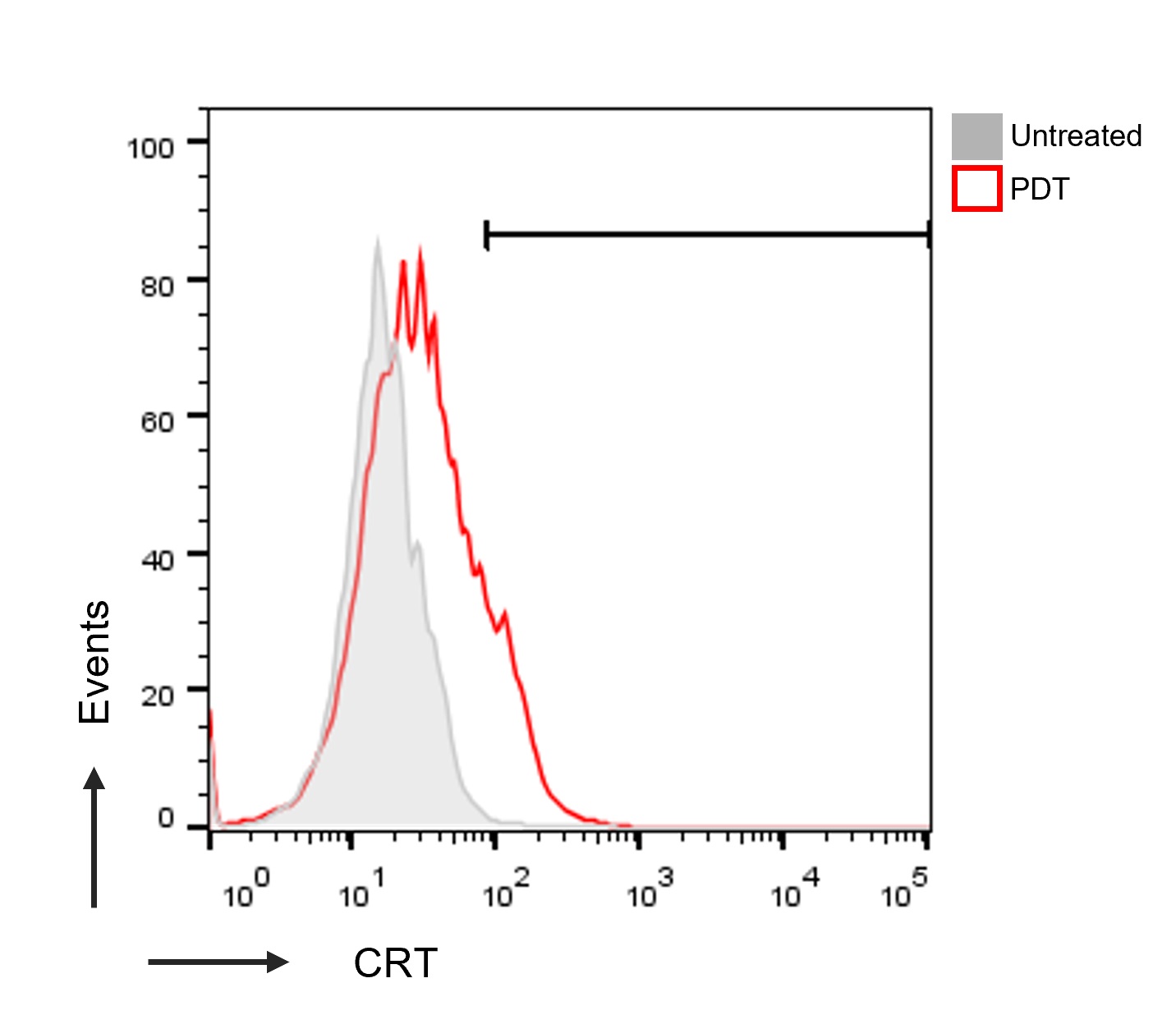

Supplement: Supplementary file 1 [file cancers-16-02568-s001.zip › Figure 4 Supp.jpg]

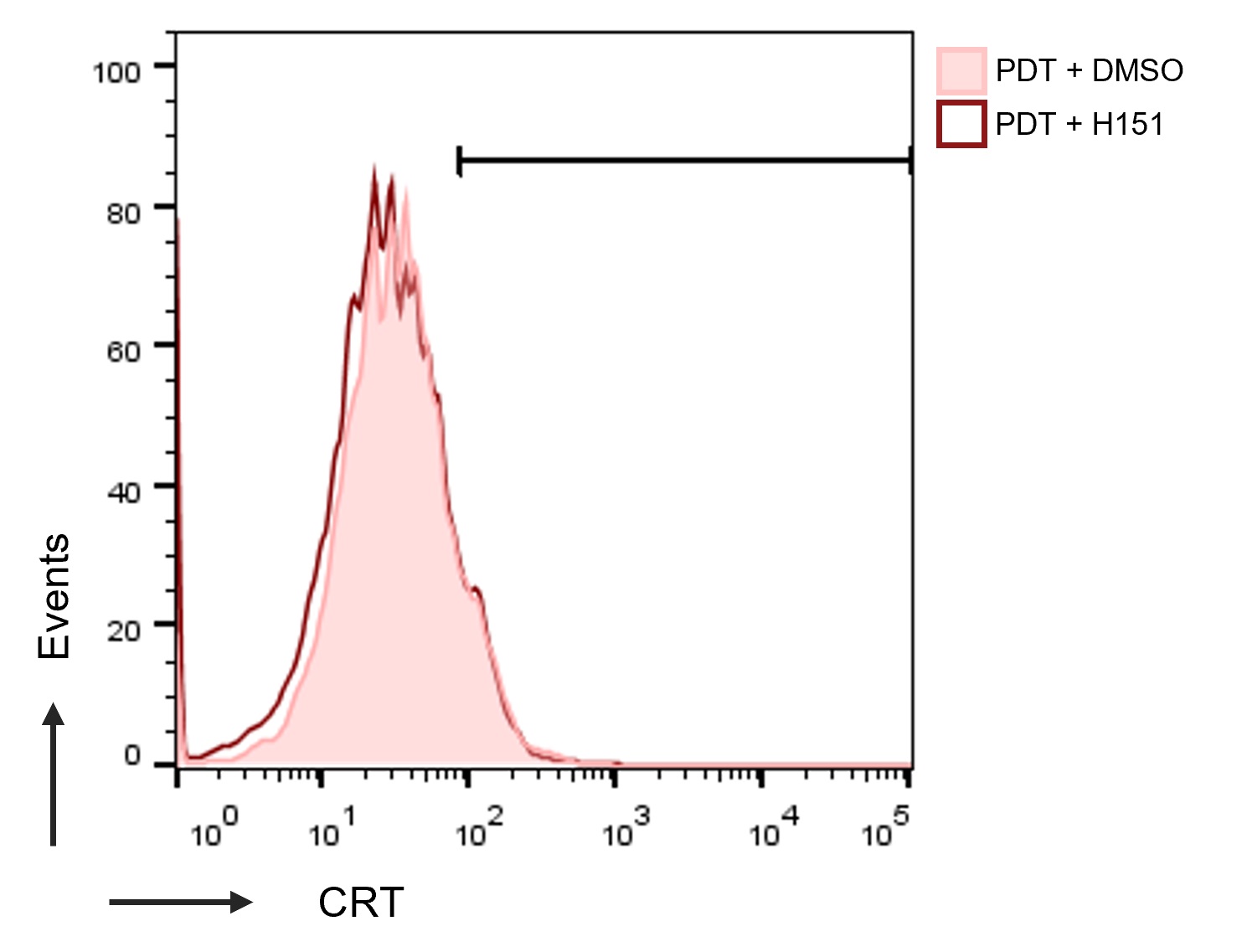

Supplement: Supplementary file 1 [file cancers-16-02568-s001.zip › Figure 5 Supp.jpg]
